# The use of fibrinolytic therapy for parapneumonic effusion in pregnancy: a case report and a review

**DOI:** 10.1186/s13019-021-01619-3

**Published:** 2021-08-21

**Authors:** Awrad Nasralla, Bryce Laing, Simon R. Turner

**Affiliations:** 1 Division of General Surgery, Department of Surgery, University of Alberta, Alberta, Canada; 2 Division of Thoracic Surgery, Department of Surgery, University of Alberta, Alberta, Canada

**Keywords:** Pregnancy, Fibrinolytics, Parapneumonic effusion

## Abstract

The use of intrapleural fibrinolytics for complicated parapneumonic effusion has been shown to be an effective and safe alternative to surgery. However, there is limited evidence about its use during pregnancy. We present a case and a review of the literature of pregnant women who had successful treatment of their complicated parapneumonic effusion with intrapleural fibrinolytics. To our knowledge this is the first review of cases of pregnant women with parapneumonic effusion managed with intrapleural fibrinolytic.

## Introduction

The incidence of pneumonia in pregnant women is similar to the general population. It is associated with serious complications such as empyema (8%), bacteremia (16%), and respiratory failure (20%). The management of complicated parapneumonic effusion consist of antibiotics, chest tube insertion, and fibrinolytics or surgery [1, 2]. The use of fibrinolytics had decreased the rate of surgical intervention for empyema from 80 to 30% [3]. However, there are no randomized controlled trials in pregnant patients to demonstrate safety of intrapleural fibrinolytics. Data from intravenous fibrinolytics for acute thrombotic events suggest that the complication rate is similar in pregnant and nonpregnant women [4]. Herein, we present a case and a literature review for cases of pregnant women with parapneumonic effusion managed with intrapleural fibrinolytic therapy.

## Methods

We present a case of a 23-week pregnant patient with empyema who was managed with thoracostomy and fibrinolytic therapy. She presented while out of country with lower respiratory tract symptoms. During admission she developed septic shock and a small left pleural effusion. Initially she was treated with antibiotics and vasopressors. Once stabilized she traveled back to her home city where she was readmitted due to a progressively enlarging left pleural effusion.

A comprehensive search was performed using the Medline, Cochrane, Pubmed, and Scopus data bases. Abstracts included based on the following inclusion criteria: (1) Pregnancy, (2) Empyema or parapneumonic effusion, (3) fibrinolytic therapy. The primary outcome of interest was resolution of the effusion clinically and radiologically. Secondary outcomes included fetus condition and follow-up after delivery.

## Case presentation

A 35-year-old pregnant, gestational age of 23 weeks, presented while out of country with 10 days history of dry cough, fatigue, and fever. On presentation she had left-sided pleuritic chest pain, and was found to be hypotensive (systolic blood pressure of 70), with oxygen saturation at 92% on room air, afebrile (36.80 °C), with respiratory rate of 25/min, and heart rate of 110/min. On physical examination, she was alert, and she had decreased breath sounds on the left side. She was admitted to the intensive care unit (ICU) with a diagnosis of septic shock secondary to pneumonia. Chest x-ray (CXR) and computed tomography (CT) of the chest revealed left side pneumonia. Significant laboratory finding were WBC of 25 and blood culture showing pansensitive group A streptococcus. She was given 5L of IV fluids and she was started on norepinephrine, ceftriaxone, and azithromycin. Fetal heart rate, assessed via Doppler, was within normal limits.

**Fig. 1:**
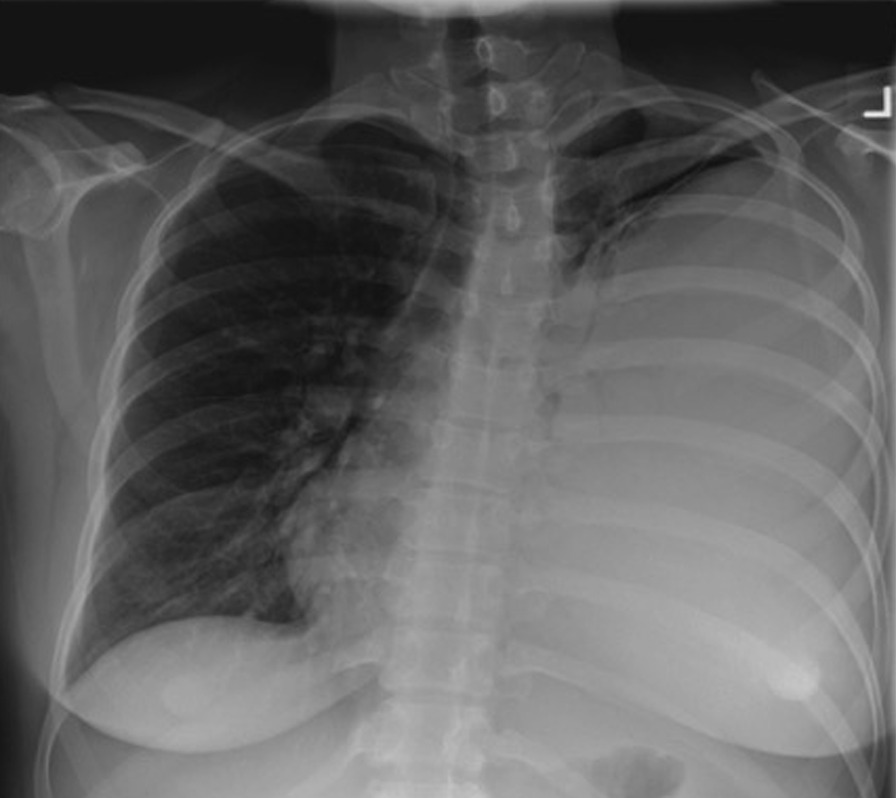
CXR showing the left pleural effusion

**Fig. 2:**
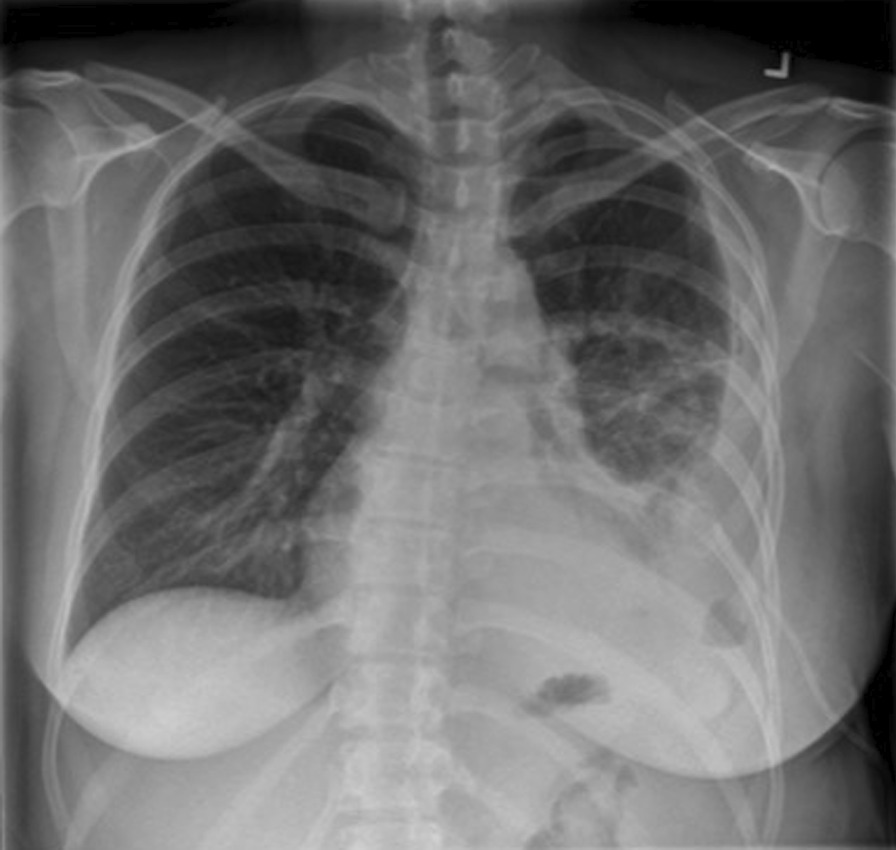
CXR following drainage of the left pleural effusion

After several days of treatment with IV ceftriaxone the patient was discharged home, with a plan to continue IV antibiotics for 2 weeks and to follow up with her family doctor. During this follow-up clinic visit, she was afebrile (36.1 °C), her blood pressure was 107/65, heart rate of 116, oxygen saturation at 95% on room air. She had reduced breath sounds over the left side of her chest. Her WBC was 25.7. Repeated blood cultures were negative. Repeated CXR revealed a large left pleural effusion with tracheal shift to the right side (Fig. 1). An 8- French pig tail was inserted under ultrasound (US) guidance to drain the left pleural effusion. Initially 240 ml of cloudy pleural fluid was drained, and pleural fluid culture was negative in the setting of ongoing treatment with an antibiotic. CXR showed incomplete resolution of left pleural effusion (Fig. 2). A multidisciplinary discussion about the advantages and disadvantage of both surgery and intrapleural thrombolytics was had with the patient. She decided to proceed with intrapleural fibrinolytics, accepting the potential risk of bleeding and possible fetal complications. One dose of 5 mg of tissue plasminogen activator (tPA) diluted in 50 ml of normal saline was given, after which a significant drainage of the left pig tail was noticed. A Fetal ultrasound following the administration of intrapleural fibrinolytics was reassuring.

**Fig. 3:**
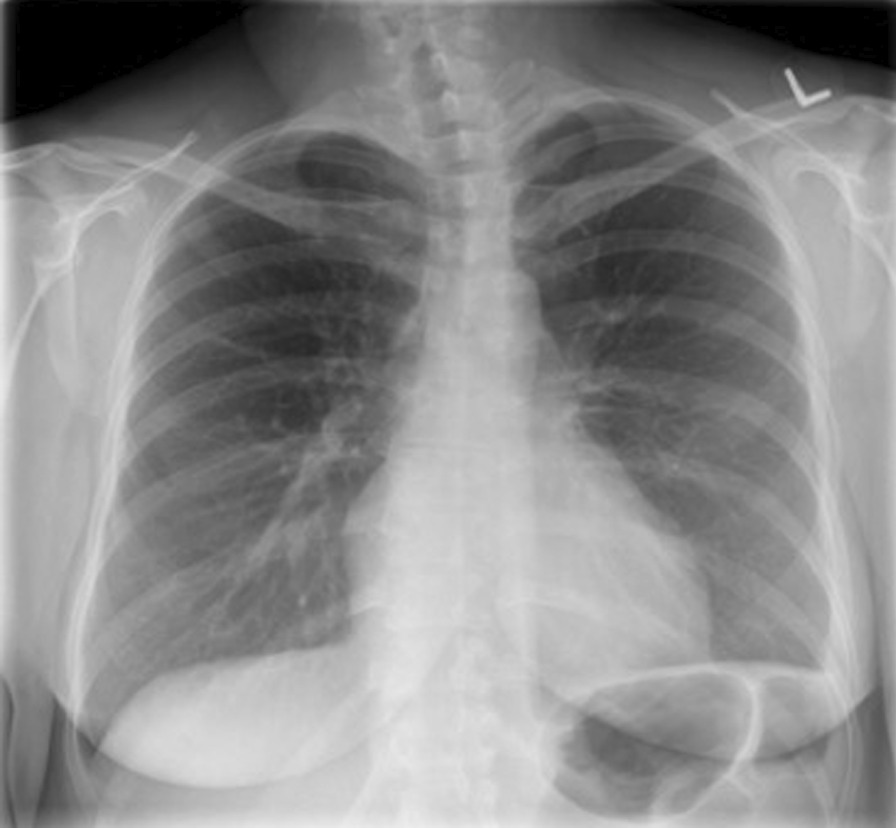
CXR showing resolution of the left plural effusion

The pigtail was removed the day after, she was discharged home with IV Penicillin G for 2 weeks, then she was switched to Amoxicillin to complete a total of 4 weeks of treatment. Her WBC normalized. Her follow up CXR showed near complete resolution of the pleural effusion. However, a repeated CXR after 6 months showed complete resolution of the effusion, and minimal residual pleural thickening in the left costophrenic angle (Fig. 3). She a had a healthy term baby via vaginal delivery.

## Results

There are 5 reported cases of treatment of complex pleural effusions in pregnant women using fibrinolytics in the literature. Mean age was 31.2 years, mean gestational age was 28.2 weeks. Management consisted of antibiotic, thoracostomy, and intrapleural fibrinolytics. All had resolution of the symptoms and pleural effusion with avoidance of surgery. Images utilized were CXR, CT scan, or magnetic resonance imaging (MRI). In all the cases the fetus status was assessed with sonography or continuous fetal cardiotocography. No complications in the form of bleeding or other morbidity to the pregnant woman nor her baby was reported.

The most commonly used fibrinolytics were streptokinase or alteplase, and the doses of fibrinolytic therapy used varied among the cases reported (Table 1).

**Table 1:** Cases of complicated parapneumonic effusion in pregnant ladies managed with intrapleural fibrinolytic therapy

Publication year	Author	Age	GA	Fibrinolytic therapy
2009	Samuel Nir [12]	24	33	Streptokinase (250,000 U/Q 8 h), total 9 doses over 72 h
2012	Hakki Ulutas [1]	22	24	Streptokinase 250,000 U/day, 2 doses
2012	Hakki Ulutas [1]	39	29	Streptokinase 250,000 U/day, 4 doses
2017	Heather Torbic [9]	35	32	2.5 mg of tPA, 1 dose
2019	Diana Amariei [8]	32	28	Two doses:2.5 mg of alteplase and 5 mg of dornase, third dose of alteplase of 5 mg
2021	Awrad Nasralla	35	23	5 mg of tPA, 1 dose

## Discussion

The use of intrapleural fibrinolytic therapy for complicated parapneumonic effusion during pregnancy is based on case reports [6]. It has been documented that the use of systematic fibrinolytics for thromboembolic disease in pregnant women was not associated with significant adverse events in comparison to general population [4]. In addition, several factors make intrapleural fibrinolytics attractive for complicated parapneumonic effusion. Those include the lower dose used for intrapleural treatment, short half-life, large molecular size with low systemic absorption, and no reported teratogenic effects on humans [7, 8]. However, side effects may be encountered such fever, allergic reactions, chest pain, and increased risk of bleeding. Of those the most worrisome is the pleural bleeding although it is uncommon, and can often be detected immediately by looking at the chest tube output. The risk of bleeding is higher in patients on anticoagulation, or those with coagulopathic disorders or renal failure [3, 9, 10].

This warrants a thorough discussion with patient about the potential adverse events. A multidisciplinary approach is recommended including obstetrics, pulmonology, infectious disease and thoracic surgery teams. In terms of appropriate dose of intrapleural fibrinolytics, we think that should be individualized to each case based on the size of the effusion, and the presence of loculation. It is worth mentioning that some cases reported the use intrapleural streptokinase to treat complicated parapneumonic effusion with success without adverse events on newborn [11].

## Conclusion

Management of parapneumonic effusion during pregnancy using fibrinolytic therapy is based on case reports. It seems to be a safe and effective alternative to surgery. A multidisciplinary approach is recommended, with early referral to thoracic surgery. The crucial aspect is the discussion between the patient and the medical team outlining the possible risk and benefits.

## Data Availability

All the data are presented.

## Abbreviations

tPA: Tissue plasminogen activator, alteplase
CT: Computed tomography
MRI: Magnetic resonance imaging
CXR: Chest X-ray
ICU: Intensive Care Unit
WBC: White blood cell count
